# Associations of different type of physical activity with all-cause mortality in hypertension participants

**DOI:** 10.1038/s41598-024-58197-2

**Published:** 2024-03-29

**Authors:** Chenliang Ge, Binghua Long, Qingjian Lu, Zhiyuan Jiang, Yan He

**Affiliations:** Department of Cardiology, The First Affiliated Hospital of Guangxi Medical University, No. 6, Shuangyong Road, Qingxiu District, Nanning, 530021 Guangxi Zhuang Autonomous Region China

**Keywords:** Hypertension, Moderate physical activity, Vigorous physical activity, Mortality, Cardiology, Hypertension

## Abstract

Few studies explored the association of different type of physical activity with all-cause mortality in hypertension (HBP) participants. A retrospective cohort analysis was performed using National Health and Nutrition Examination Survey (NHANES) data to explore association of moderate-intensity physical activity (MPA), vigorous-intensity physical activity (VPA), sedentary behavior with mortality in HBP individuals. Among 10,913 HBP participants followed for a median of 6.2 years, VPA was not associated with a reduction in all-cause mortality compared to participants without VPA in multivariate Cox survival analysis. MPA was linked to lower all-cause mortality at durations of 0–150 min/week (HR, 0.72; 95% CI 0.58–0.88), 150–300 min/week (HR, 0.71; 95% CI 0.52–0.96), and > 300 min/week (HR, 0.61; 95% CI 0.49–0.77) compared to no MPA. Sedentary behavior of 6–8 h/day (HR, 1.35; 95% CI 1.15–1.59) and > 8 h/day (HR, 1.55; 95% CI 1.34–1.79) were associated with increased mortality risk versus < 6 h/day. Further research is needed to explore whether VPA can improve outcomes for HBP individuals and to determine the optimal duration of VPA.MPA is linked to lower mortality risk, indicating its potential as the best physical activity intensity for HBP individuals.

## Introduction

Substantial research underscores the value of physical activity in diminishing mortality in adult population^1,2^. Exercise plays a pivotal role in managing hypertension (HBP), a significant risk factor for cardiovascular disease and a key contributor to cardiovascular mortality. HBP is strongly linked to a sedentary lifestyle and physical activity, including both aerobic and resistance exercises, has been shown to effectively delay the onset of HBP and reduce blood pressure (BP)^3,4^. The World Health Organization's guidelines advocate for a weekly engagement of 150–300 min in moderate-intensity physical activity (MPA) or 75–150 min in vigorous-intensity physical activity (VPA), or a proportionate blend of both, to optimize health benefits for HBP population^5,6^. Nevertheless, there is currently insufficient evidence supporting this physical activity recommendation, the potential interactive effects of different physical activity intensities on health outcomes are not well understood^7,8^. This study aims to examine the prospective associations of MPA, VPA, sedentary behavior and their interactive effects with all-cause mortality in HBP individuals using the National Health and Nutrition Examination Survey (NHANES) data from 2007–2018 during a retrospective cohort analysis.

## Methods

### Study population

**Figure 1:**
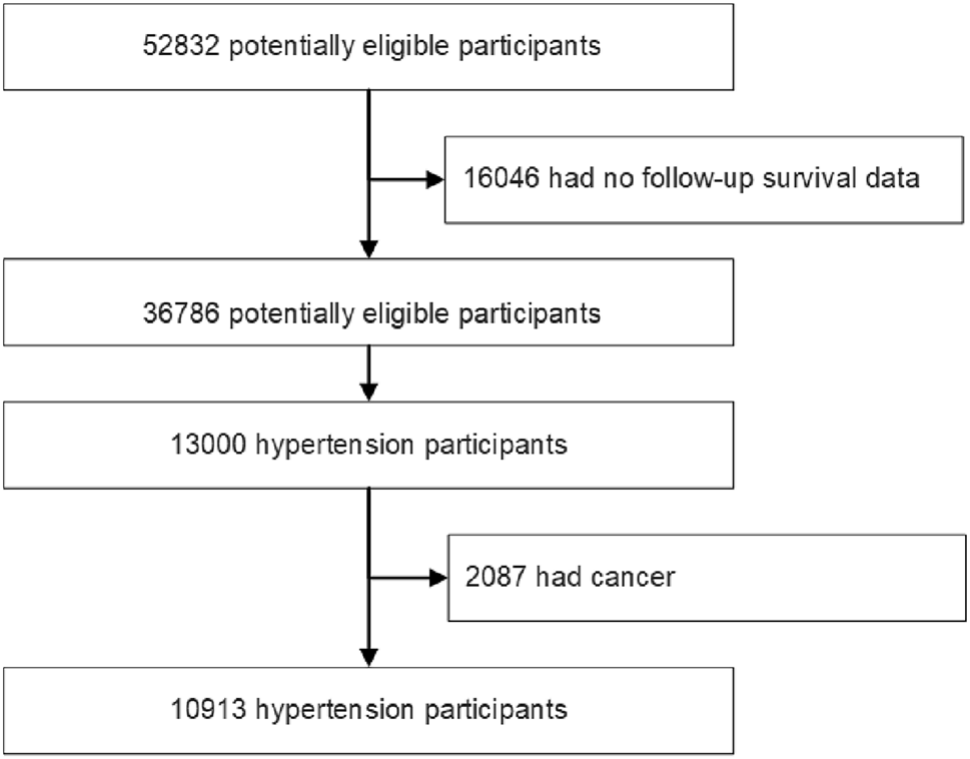
Flow diagram of study sample selection.

This study employed data obtained from NHANES, which is a detailed, multistage, stratified health survey conducted across the United States, encompassing a wide array of health-related information^9–13^. This study received ethical approval from the National Center for Health Statistics (NCHS) Institutional Review Board, and informed consent was obtained from all participants. We confirmed all methods in the present study were performed in accordance with the Declaration of Helsinki. For our analysis, we compiled a dataset from the NHANES public data files covering the years 2007–2018. We excluded individuals missing essential follow-up survival data and physical activity time. From the initial pool of 52,832 participants with physical data, a subset of 36,786 individuals with survival data was identified. Focusing specifically on HBP, we selected 13,000 participants, excluding those with existing any cancer (n = 2087), ultimately resulting in a sample of 10,913 HBP participants (Fig. 1). HBP participants are subjects who self-report having been diagnosed with HBP by a physician. The method for measuring BP is as follows: after resting quietly in a sitting position for 5 min and determining the maximum inflation level, three consecutive BP readings are obtained. If a BP measurement is interrupted or incomplete, a fourth attempt may be made, the average of the three BP readings is calculated. NCHS has established a linkage between various population surveys and death certificate records from the National Death Index. The follow-up period was calculated in person-months, starting from the interview date to the event of death, loss to follow-up, or the end of the mortality follow-up period on December 31, 2019.

### Time of physical activity

Data on physical activity time were gathered through participant responses to the Global Physical Activity Questionnaire (GPAQ). The GPAQ has been previously validated to collect information related to work activity and recreational activities^14^. During face-to-face interviews, participants were asked to detail the frequency and duration of MPA and VPA during a typical week. The time of physical activity was calculated by summing both work and recreational activities.MPA was categorized into four levels (0 min/week, 0–150 min/week, 150–300 min/week, > 300 min/week) and sedentary activity time was categorized into three levels (< 6 h/day, 6–8 h/day, > 8 h/day).

### Sociodemographic characteristics and covariates

The study participants provided a range of demographic information, including age, gender, body mass index (BMI), smoke history and racial categorization (Mexican American, Other Hispanic, Non-Hispanic White, Non-Hispanic Black and Other Race). Educational levels were categorized into four groups: less than high school, high school graduate, some college or associate degree, and college graduate. Marital status was classified into three categories: never married, married or living with a partner and separated or divorced or widowed. The ratio of family income to poverty level was categorized as < 1, 1–3, or > 3. Additionally, vital biomarkers including albumin and creatinine were obtained from the principal NHANES dataset. Pre-comorbidities were identified including diabetes mellitus (DM), coronary heart disease (CHD), heart failure (HF), hypercholesterolemia, stroke, chronic bronchitis and liver diseases. The presence of these conditions was determined based on physician diagnoses documented in the NHANES data.

### Statistical analysis

We accounted for the complex survey design of NHANES by incorporating sample weights, clustering, and stratification. To achieve nationally representative estimates, the original survey weights were adjusted and utilized in the analysis, taking into account the appropriate adjustments^15,16^. Initially, we examined the association of VPA, MPA and sedentary activity time with all-cause mortality among all HBP participants. VPA, MPA and sedentary activity were all included as covariates in the Cox survival analysis. Adjustments were made for gender, age, race, marital status, educational level, family income level, smoke history, albumin, creatinine levels and the presence of comorbidities such as CHD, stroke, chronic bronchitis, liver conditions and high cholesterol. Statistical significance was defined as *P* ≤ 0.05. In the subsequent sections, we conducted association between MPA and all-cause mortality across various subgroups defined by the presence of VPA and different durations of sedentary activity. In sensitivity analysis, we excluded patients with a follow-up duration of less than 1 year, as well as those who died due to accidental events, and re-analyzed the data accordingly. Missing data were addressed using the multiple imputation technique, with less than 3% missing values for most variables, except for BMI (5.6% missing), albumin (9.67% missing) and creatinine (9.69% missing)^17^.

### Ethical approval

This study adhered to the protocols and procedures of NHANES, all participants gave written informed consent at the time of study enrollment, ensuring the ethical compliance of the research design and conduct.

## Results

### Patient characteristics

The study encompassed a total of 10,913 participants with HBP, accounting for 70,303 person-years, with a median follow-up duration of 6.2 years (interquartile range, IQR: 3.4–9.3). Out of these, 1715 participants succumbed to all causes, including 488 cardiovascular disease (CVD) deaths and 298 cancer deaths. Participants were divided into two groups: those with VPA (N = 2746) and those without VPA (N = 8167). Compared to the non-VPA group, the VPA group was characterized by a younger age, lower BMI, a higher proportion of males, and a greater percentage of individuals with an education level of high school or above. Furthermore, the VPA group had lower rates of comorbidities including HF, CHD, diabetes, stroke, hypercholesterolemia, chronic bronchitis and liver diseases. Additionally, participants in the VPA group engaged in more minutes per week of MPA with a higher proportion of 0–150 min/week, 150–300 min/week, and > 300 min/week compared to those in the non-VPA group. The duration of sedentary activity was shorter in the VPA group with a higher proportion of participants spending less than 6 h/day sedentary and lower proportions spending 6–8 h/day or more than 8 h/day sedentary. Table 1 summarizes the baseline sample characteristics stratified by presence of VPA (Table 1).

**Table 1:** Baseline characteristics of HBP participants stratified by whether had VPA from NHANES.

Characteristics	Types of physical activity		*P* value
	No VPA	Had VPA	
Estimated N	42,846,457	18,511,784	
N	8167	2746	
Age (year)	58.82 (14.28)	49.56 (14.48)	< 0.001
BMI (kg/m^2^)	31.85 (7.67)	30.86 (6.53)	< 0.001
SBP (mmHg)	132.19 (19.91)	129.82 (17.75)	< 0.001
DBP (mmHg)	71.62 (14.40)	75.21 (13.34)	< 0.001
Gender (%)			
Male	40.2%	67.1%	< 0.001
Female	59.8%	32.9%	
Race/ethnicity			
Mexican American	6.3%	6.8%	0.205
Other hispanic	5.2%	5.3%	
Non hispanic white	64.5%	66.0%	
Non hispanic black	16.4%	14.2%	
Other race	7.6%	7.6%	
Education level			
Less than high school	22.1%	13.4%	< 0.001
High school	26.1%	24.5%	
Some college or associate degree	31.3%	34.6%	
College graduate or above	20.5%	27.4%	
Married state			
Never married	9.5%	13.7%	< 0.001
Married/living with partner	60.9%	66.2%	
Widowed/divorced/separated	29.6%	20.1%	
Smoke history			
Ever	48.5%	51.0%	0.093
Never	51.5%	49.0%	
Ratio of family income to poverty level			
< 1	15.5%	11.4%	< 0.001
1–3	46.5%	40.3%	
> 3	38.0%	48.3%	
Pre-exist comorbidities			
HF (%)			
Yes	6.9%	2.7%	< 0.001
No	93.1%	97.3%	
CHD (%)			
Yes	8.3%	5.2%	< 0.001
No	91.7%	94.8%	
Stroke (%)			
Yes	8.0%	2.9%	< 0.001
No	92.0%	97.1%	
Diabetes (%)			
Yes	24.4%	13.7%	< 0.001
No	75.6%	86.3%	
High cholesterol (%)			
Yes	55.1%	45.9%	< 0.001
No	44.9%	54.1%	
Chronic bronchitis (%)			
Yes	9.3%	6.5%	0.004
No	90.7%	93.5%	
Liver condition (%)			
Yes	6.1%	5.4%	0.549
No	93.9%	94.5%	
Laboratory test			
Creatinine (mg/dL)	0.97 (0.61)	0.94 (0.37)	0.023
Albumin (g/dL)	4.16 (0.35)	4.28 (0.34)	< 0.001
MPA time			
0 min/week	52.3%	15.3%	< 0.001
0–150 min/week	18.4%	18.9%	
150–300 min/week	10.6%	15.2%	
> 300 min/week	18.7%	50.6%	
Sedentary activity time			
< 6 h/day	52.6%	65.1%	< 0.001
6–8 h/day	20.6%	15.0%	
> 8 h/day	26.8%	20.0%	

All estimates accounted for complex survey designs. All numbers in the table are weighted percentages or means. *HBP* hypertension, *SBP* systolic blood pressure, *DBP*, diastolic blood pressure, *VPA* vigorous-intensity physical activity, *MPA* moderate-intensity physical activity, *NHANES* National Health and Nutrition Examination Survey, *BMI* body-mass index, *DM* diabetes, *HF* heart failure. *CHD* coronary heart disease.

### Association of physical category with all-cause mortality

In univariate Cox survival analysis, VPA was associated with a reduction in all-cause mortality compared to participants without VPA (Hazard Ratio [HR], 0.32; 95% Confidence Interval [CI], 0.25–0.41). MPA durations of 0–150 min/week (HR, 0.48; 95% CI 0.39–0.58), 150–300 min/week (HR, 0.46; 95% CI 0.36–0.59), and > 300 min/week (HR, 0.32; 95% CI 0.26–0.39) were associated with a reduction in all-cause mortality compared to participants without MPA. Sedentary activity times of 6–8 h/day (HR, 1.49; 95% CI 1.24–1.79) and > 8 h/day (HR, 1.51; 95% CI 1.29–1.78) were associated with an increase in all-cause mortality compared to < 6 h/day. In Model 1 adjusted for VPA, MPA, sedentary activity time, gender, age, race, marital status, educational level, family income level, smoke history, VPA was associated with a reduction in all-cause mortality compared to participants without VPA (HR, 0.73; 95% CI 0.56–0.95). MPA durations of 0–150 min/week (HR, 0.69; 95% CI 0.56–0.85), 150–300 min/week (HR, 0.69; 95% CI 0.53–0.91), and > 300 min/week (HR, 0.57; 95% CI 0.46–0.71) were associated with a reduction in all-cause mortality compared to participants without MPA. Sedentary activity times of 6–8 h/day (HR, 1.41; 95% CI 1.18–1.66) and > 8 h/day (HR, 1.63; 95% CI 1.41–1.90) were associated with an increase in all-cause mortality compared to < 6 h/day. In Model 2 adjusted for VPA, MPA, sedentary activity time, gender, age, race, marital status, educational level, family income level, smoke history, albumin, creatinine levels and the presence of comorbidities including CHD, stroke, chronic bronchitis, liver conditions and high cholesterol, VPA was not associated with a reduction in all-cause mortality compared to participants without VPA (HR, 0.81; 95% CI 0.61–1.06). MPA durations of 0–150 min/week (HR, 0.72; 95% CI 0.58–0.88), 150–300 min/week (HR, 0.71; 95% CI 0.52–0.96), and > 300 min/week (HR, 0.61; 95% CI 0.49–0.77) were associated with a reduction in all-cause mortality compared to participants without MPA. Sedentary activity times of 6–8 h/day (HR, 1.35; 95% CI 1.15–1.59) and > 8 h/day (HR, 1.55; 95% CI 1.34–1.79) were associated with an increase in all-cause mortality compared to < 6 h/day (Table 2). In sensitivity analyses, where participants with follow-up events less than 1 year and those who died from accidental causes were excluded, leaving 10,718 subjects, the results were similar to the primary analysis (Supplementary table).

**Table 2:** Estimated association of VPA, MPA and recreational time with all-cause mortality in HBP participants.

Character	Time	Participants N	Person-years	Events N (%)	Unadjusted			Model 1			Model 2		
					HR	95% CI	*P *value	HR	95% CI	*P *value	HR	95% CI	*P *value
VPA	No	8167	52,768	1540 (18.9%)	Ref			Ref			Ref		
	Yes	2746	17,535	175 (6.4%)	0.32	0.25–0.41	< 0.001	0.73	0.56–0.95	0.023	0.81	0.61–1.06	0.124
MPA (min/wk)	0	5220	33,318	1138 (21.8%)	Ref			Ref			Ref		
	0–150	1861	11,945	229 (12.3%)	0.48	0.39–0.58	< 0.001	0.69	0.56–0.85	< 0.001	0.72	0.58–0.88	0.001
	150–300	1187	7684	127 (10.7%)	0.46	0.36–0.59	< 0.001	0.69	0.53–0.91	< 0.001	0.71	0.52–0.96	0.025
	> 300	2645	17,356	221 (8.4%)	0.32	0.26–0.39	< 0.001	0.57	0.46–0.71	< 0.001	0.61	0.49–0.77	< 0.001
Recreational time (hour/day)	< 6	6571	44,194	899 (13.7%)	Ref			Ref			Ref		
	6–8	2054	12,580	378 (18.4%)	1.49	1.24–1.79	< 0.001	1.41	1.18–1.66	< 0.001	1.35	1.15–1.59	< 0.001
	> 8	2288	13,529	438 (19.1%)	1.51	1.29–1.78	< 0.001	1.63	1.41–1.90	< 0.001	1.55	1.34–1.79	< 0.001

Survey sample weights were taken into consideration in the Cox models accompanying the NHANES data. Covariates in Model 1 included VPA, MPA, recreational time, gender, age, race, marital status, educational level, family income level, smoke history. Model 2 also included albumin, creatinine levels and the presence of comorbidities including CHD, stroke, chronic bronchitis, liver conditions and high cholesterol. *HBP* hypertension, *VPA*, vigorous-intensity physical activity, *MPA* moderate-intensity physical activity.

In participants without VPA, MPA durations of 0–150 min/week (HR, 0.59; 95% CI 0.51–0.69), 150–300 min/week (HR, 0.54; 95% CI 0.44–0.66), and > 300 min/week (HR, 0.47; 95% CI 0.39–0.56) were all associated with lower all-cause mortality compared to those without MPA. Among participants with VPA, only MPA > 300 min/week (HR, 0.62; 95% CI 0.42–0.91) was associated with lower all-cause mortality compared to those without MPA. There was no interaction between MPA and VPA (*P* value for interaction: 0.213). There was interaction between MPA and sedentary activity (*P* value for interaction: < 0.001). Among participants with sedentary activity time < 6 h/day, MPA durations of 0–150 min/week (HR, 0.74; 95% CI 0.62–0.91), 150–300 min/week (HR, 0.69; 95% CI 0.55–0.87), and > 300 min/week (HR, 0.45; 95% CI 0.37–0.54) were all associated with reduced mortality rates. For those with sedentary activity time of 6–8 h/day, MPA durations of 0–150 min/week (HR, 0.55; 95% CI 0.41–0.75), 150–300 min/week (HR, 0.44; 95% CI 0.29–0.66), and > 300 min/week (HR, 0.35; 95% CI 0.25–0.50) were all associated with reduced mortality rates. In participants with sedentary activity time > 8 h/day, MPA durations of 0–150 min/week (HR, 0.31; 95% CI 0.22–0.43), 150–300 min/week (HR, 0.17; 95% CI 0.09–0.31), and > 300 min/week (HR, 0.32; 95% CI 0.22–0.47) were all associated with reduced mortality rates (Table 3).

**Table 3:** Subgroup analysis for association of MPA with all-cause mortality in HBP participants.

Characrer	MPA (min/wk)	Participants N	Person-years	Events N (%)	HR	95% CI	*P* value	*P *value for interaction
VPA								0.213
No	0	4744	30,156	1097 (23.1%)	Ref			
	0–150	1346	8768	189 (14.0%)	0.59	0.51–0.69	< 0.001	
	150–300	782	5194	104 (13.3%)	0.54	0.44–0.66	< 0.001	
	> 300	1295	8650	150 (11.6%)	0.47	0.39–0.56	< 0.001	
Yes	0	476	3161	41 (8.6%)	Ref			
	0–150	515	3178	40 (7.8%)	0.99	0.64–1.53	0.968	
	150–300	405	2490	23 (5.7%)	0.92	0.43–1.21	0.214	
	> 300	1350	8706	71 (5.3%)	0.62	0.42–0.91	0.015	
Sedentary activity time(hour/day)								< 0.001
< 6	0	2860	19,438	518 (18.1%)	Ref			
	0–150	1032	6822	135 (13.1%)	0.74	0.62–0.91	0.003	
	150–300	735	4803	88 (12.0%)	0.69	0.55–0.87	0.002	
	> 300	1944	13,131	158 (8.1%)	0.45	0.37–0.54	< 0.001	
6–8	0	1064	6444	265 (24.9%)	Ref			
	0–150	359	2201	50 (13.9%)	0.55	0.41–0.75	< 0.001	
	150–300	228	1463	27 (11.8%)	0.44	0.29–0.66	< 0.001	
	> 300	403	2472	36 (8.9%)	0.35	0.25–0.50	< 0.001	
> 8	0	1296	7436	355 (27.4%)	Ref			
	0–150	470	2922	44 (9.4%)	0.31	0.22–0.43	< 0.001	
	150–300	224	1418	12 (5.4%)	0.17	0.09–0.31	< 0.001	
	> 300	298	1753	27 (9.1%)	0.32	0.22–0.47	< 0.001	

Survey sample weights were taken into consideration in the Cox models accompanying the NHANES data. *HBP* hypertension, *VPA*, vigorous-intensity physical activity, *MPA*, moderate-intensity physical activity.

## Discussion

In our population-based study, we explored the correlation between different durations of VPA, MPA, sedentary activity and mortality among participants with HBP, utilizing data from NHANES, a nationally representative sample. Longer duration of MPA and shorter duration of sedentary activity were associated with a decrease in mortality in both univariate and multivariate Cox analyses. VPA was associated with a reduction in all-cause mortality compared to participants without VPA in univariate Cox survival analysis. As compared to the participants without VPA, the participants with VPA were characterized by a younger age, lower BMI, a higher proportion of males, and a greater percentage of individuals with an education level of high school or above. Furthermore, participants with VPA had lower rates of comorbidities including HF, CHD, diabetes, stroke, hypercholesterolemia, chronic bronchitis and liver diseases. In the multivariate Cox proportional hazards analysis, considering the effects of MPA, sedentary activity and other covariates simultaneously, we found that the presence of VPA was not associated with a reduction in all-cause mortality.

Vigorous-intensity physical activity has been shown to have a beneficial impact on the prognosis and mortality of HBP patients, but there are also different opinions or controversies in some studies. A study reported that compared with the least active group, 75–150 min/week of VPA or more was associated with few further benefits, even weakening the cardiovascular benefits. A relatively short duration of VPA was probably more beneficial than a longer duration of VPA^7^. Two recent analyses indicate reductions in mortality across the general populace could potentially be attained with VPA quantities less than those presently advised, yet neither study delved into the impact of prolonged VPA exposure^18,19^. The findings suggest that minimal VPA levels might confer greater benefits, hinting that the ideal VPA dosage may fall below current guidelines. VPA may trigger a short-term surge in BP, with HBP individuals possibly experiencing an exaggerated BP reaction to physical activity^20^. The propensity for atherosclerosis formation escalates in HBP patients, where sudden BP spikes could provoke the rupture of atherosclerotic plaques and lead to acute arterial thrombosis, thereby heightening the risk of cardiovascular incidents^6,21^. These observations imply the necessity of a more judicious approach to physical activity recommendations for those with HBP.

A substantial body of research has confirmed that longer durations of MPA and shorter durations of sedentary activity are associated with a reduction in all-cause mortality among HBP patients^22–25^. Our study results are in line with previous research, and we discovered some interesting findings in our subgroup analyses. In the subgroup without VPA, longer durations of MPA were still associated with a reduction in all-cause mortality among HBP patients. However, in the subgroup with VPA, longer durations of MPA were almost not associated with lower all-cause mortality, with only durations > 300 min/week showing statistical significance. We speculate that the possible reason is that participants engaging in VPA generally have better physical function and exercise capacity, with fewer comorbidities such as HF, myocardial infarction and chronic bronchitis, which impact lifespan. Therefore, in a population with generally longer survival, different durations of MPA did not show differences, and statistical analysis was also deemed meaningless.

In the subgroups with varying durations of sedentary activity, we observed that individuals with longer sedentary activity times benefited more from MPA. From the HR, the benefits were greatest in the subgroup with more than 8 h per day of sedentary activity, followed by the 6–8 h per day subgroup, and were least in the subgroup with less than 6 h per day of sedentary activity. This suggests that individuals with longer durations of sedentary activity could improve their prognosis by engaging in MPA. MPA can effectively counteract the adverse effects of prolonged sedentary activity, thereby yielding benefits. Studies collectively support the notion that engaging in MPA is a viable and effective approach to mitigating the negative health impacts of prolonged sedentary behavior^26–28^. It’s also important to acknowledge that shorter sedentary times may imply higher total physical activity, influencing outcomes, suggesting that the mortality benefits associated with MPA could also reflect a generally more active lifestyle.

Several limitations should be acknowledged. We did not explore the impact of different types of exercise on BP levels. Time of MPA and VPA were assessed using a single self-reported measure, which is susceptible to recall bias and potential differential misclassification. Information on medication treatment for patients with HBP was not collected. The observational nature of the study imposes limitations related to residual confounding, necessitating cautious interpretation of the associations as indicative rather than causal. The relationship between VPA and all-cause mortality still needs further exploration, as our study is retrospective and cannot establish causality. Furthermore, our study classified VPA based on its presence rather than varying durations, leaving the relationship between different lengths of VPA and all-cause mortality unclear.

Our findings underscore the association of MPA with reduced all-cause mortality among individuals with HBP. Notably, VPA did not show a direct association with mortality reduction. Our study also revealed an interaction between MPA and sedentary behavior, indicating that the benefits of MPA are pronounced in those with longer time of sedentary behavior. These findings suggest that promoting MPA, alongside strategies to reduce sedentary time, could be a crucial component of public health recommendations and clinical guidelines for managing HBP and enhancing longevity.

## Conclusions

MPA is linked to lower mortality risk, indicating its potential as the best physical activity intensity for HBP individuals. Further research is needed to explore whether VPA can improve outcomes for HBP individuals and to determine the optimal duration of VPA.

### Supplementary Information

Supplementary Table 1.

## Data Availability

All National Health and Nutrition Examination Survey data were accessed from https://www.cdc.gov/nchs/nhanes.htm.

## References

[1] Gebel K, Ding D, Chey T, Stamatakis E, Brown WJ, Bauman AE (2015). Effect of moderate to vigorous physical activity on all-cause mortality in middle-aged and older Australians. JAMA Int. Med..

[2] Saint-Maurice PF, Coughlan D, Kelly SP, Keadle SK, Cook MB, Carlson SA, Fulton JE, Matthews CE (2019). Association of leisure-time physical activity across the adult life course with all-cause and cause-specific mortality. JAMA Netw. Open.

[3] Laukkanen JA, Kunutsor SK (2021). Fitness and reduced risk of hypertension—approaching causality. J. Hum. Hypertens..

[4] Dimeo F, Pagonas N, Seibert F, Arndt R, Zidek W, Westhoff TH (2012). Aerobic exercise reduces blood pressure in resistant hypertension. Hypertension.

[5] Bull FC, Al-Ansari SS, Biddle S, Borodulin K, Buman MP, Cardon G, Carty C, Chaput J-P, Chastin S, Chou R (2020). World Health Organization 2020 guidelines on physical activity and sedentary behaviour. Br. J. Sport. Med..

[6] Williams B, Mancia G, Spiering W, Agabiti Rosei E, Azizi M, Burnier M, Clement DL, Coca A, de Simone G, Dominiczak A (2018). 2018 ESC/ESH Guidelines for the management of arterial hypertension. Euro. Heart J..

[7] Xiang B, Zhou Y, Wu X, Zhou X (2023). Association of device-measured physical activity with cardiovascular outcomes in individuals with hypertension. Hypertension.

[8] Ekelund U, Tarp J, Fagerland MW, Johannessen JS, Hansen BH, Jefferis BJ, Whincup PH, Diaz KM, Hooker S, Howard VJ (2020). Joint associations of accelero-meter measured physical activity and sedentary time with all-cause mortality: A harmonised meta-analysis in more than 44 000 middle-aged and older individuals. Br. J. Sport. Med..

[9] Curtin LR, Mohadjer LK, Dohrmann SM, Montaquila JM, Kruszan-Moran D, Mirel LB, Carroll MD, Hirsch R, Schober S, Johnson CL (2012). The national health and nutrition examination survey: Sample design, 1999–2006. Vital and health statistics series 2. Data Eval. Method. Res..

[10] Curtin LR, Mohadjer LK, Dohrmann SM, Kruszon-Moran D, Mirel LB, Carroll MD, Hirsch R, Burt VL, Johnson CL (2013). National health and nutrition examination survey: Sample design, 2007–2010. Vital and health statistics series 2. Data Eval. Method. Res..

[11] Johnson CL, Dohrmann SM, Burt VL, Mohadjer LK (2014). National health and nutrition examination survey: Sample design, 2011–2014. Vital and health statistics series 2. Data Eval. Method. Res..

[12] Chen TC, Clark J, Riddles MK, Mohadjer LK, Fakhouri THI (2020). National health and nutrition examination survey, 2015–2018: Sample design and estimation procedures. Vital and health statistics series 2. Data Eval. Method. Res..

[13] Patel CJ, Pho N, McDuffie M, Easton-Marks J, Kothari C, Kohane IS, Avillach P (2016). A database of human exposomes and phenomes from the US national health and nutrition examination survey. Sci. Data.

[14] Arem H, Pfeiffer RM, Engels EA, Alfano CM, Hollenbeck A, Park Y, Matthews CE (2015). Pre- and postdiagnosis physical activity, television viewing, and mortality among patients with colorectal cancer in the national institutes of health-AARP diet and health study. J. Clin. Oncol..

[15] Stierman, B. *et al.* National Health and Nutrition Examination Survey 2017–March 2020 prepandemic data files development of files and prevalence estimates for selected health outcomes (2021). 10.15620/cdc:106273 PMC11513744 39380201

[16] Johnson CL, Paulose-Ram R, Ogden CL, Carroll MD, Kruszon-Moran D, Dohrmann SM, Curtin LR (2013). National health and nutrition examination survey: analytic guidelines, 1999–2010. Vital and health statistics Series 2. Data Eval. Method. Res..

[17] Pan S, Chen S (2023). Empirical comparison of imputation methods for multivariate missing data in public health. Int. J. Environ. Res. Public Health.

[18] Ahmadi MN, Clare PJ, Katzmarzyk PT, Del Pozo CB, Lee IM, Stamatakis E (2022). Vigorous physical activity, incident heart disease, and cancer: How little is enough?. Eur. Heart J..

[19] Stamatakis E, Ahmadi MN, Gill JMR, Thøgersen-Ntoumani C, Gibala MJ, Doherty A, Hamer M (2022). Association of wearable device-measured vigorous intermittent lifestyle physical activity with mortality. Nat. Med..

[20] Börjesson M, Onerup A, Lundqvist S, Dahlöf B (2016). Physical activity and exercise lower blood pressure in individuals with hypertension: Narrative review of 27 RCTs. Br. J. Sport. Med..

[21] Stone PH, Libby P, Boden WE (2023). Fundamental pathobiology of coronary atherosclerosis and clinical implications for chronic ischemic heart disease management-the plaque hypothesis: A narrative review. JAMA Cardiol..

[22] Jabbarzadeh Ganjeh, B. *et al.* Effects of aerobic exercise on blood pressure in patients with hypertension: A systematic review and dose-response meta-analysis of randomized trials. *Hypertens. Res.* **47**(2), 385–398(2023). 10.1038/s41440-023-01467-9 37872373

[23] Carnethon MR, Evans NS, Church TS, Lewis CE, Schreiner PJ, Jacobs DR, Sternfeld B, Sidney S (2010). Joint associations of physical activity and aerobic fitness on the development of incident hypertension: Coronary artery risk development in young adults. Hypertension..

[24] Alpsoy Ş (2020). Exercise and hypertension. Adv. Exp. Med. Biol..

[25] Juraschek SP, Blaha MJ, Whelton SP, Blumenthal R, Jones SR, Keteyian SJ, Schairer J, Brawner CA, Al-Mallah MH (2014). Physical fitness and hypertension in a population at risk for cardiovascular disease: The Henry Ford exercise testing (FIT) project. J. Am. Heart Assoc..

[26] Bassuk SS, Manson JE (2005). Epidemiological evidence for the role of physical activity in reducing risk of type 2 diabetes and cardiovascular disease. J. Appl. Physiol..

[27] Cerqueira É, Marinho DA, Neiva HP, Lourenço O (2019). Inflammatory effects of high and moderate intensity exercise-a systematic review. Front. Physiol..

[28] O'Donovan G, Kearney EM, Nevill AM, Woolf-May K, Bird SR (2005). The effects of 24 weeks of moderate-or high-intensity exercise on insulin resistance. Eur. J. Appl. Physiol..

