# Supplementary material for: Associations of different type of physical activity with all-cause mortality in hypertension participants
Source: Sci Rep. 2024 Mar 29;14:7515. doi: 10.1038/s41598-024-58197-2 (PMC10980699; doi:10.1038/s41598-024-58197-2)
Supplement: Supplementary file 1 — Supplementary Table 1. [file 41598_2024_58197_MOESM1_ESM.docx]

Estimated association of VPA, MPA and sedentary activity time with all-cause mortality in HBP participants in Sensitivity Analyses

| **Character** | **Time** | **Participants N** | **Person-years** | **Events N(%)** |  | **Unadjusted** | | |  | **Model 1** | | |  | **Model 2** | | |
| --- | --- | --- | --- | --- | --- | --- | --- | --- | --- | --- | --- | --- | --- | --- | --- | --- |
|  |  |  |  |  |  | **HR** | **95% CI** | ***P* Value** |  | **HR** | **95% CI** | ***P* Value** |  | **HR** | **95% CI** | ***P* Value** |
| **VPA** | No | 8167 | 52768 | 1540(18.9%) |  | Ref |  |  |  | Ref |  |  |  | Ref |  |  |
|  | Yes | 2746 | 17535 | 175(6.4%) |  | 0.32 | 0.25~0.41 | <0.001 |  | 0.73 | 0.56~0.95 | 0.023 |  | 0.81 | 0.61~1.06 | 0.124 |
|  |  |  |  |  |  |  |  |  |  |  |  |  |  |  |  |  |
| **MPA(min/wk)** | 0 | 5220 | 33318 | 1138(21.8%) |  | Ref |  |  |  | Ref |  |  |  | Ref |  |  |
|  | 0~150 | 1861 | 11945 | 229(12.3%) |  | 0.48 | 0.39~0.58 | <0.001 |  | 0.69 | 0.56~0.85 | <0.001 |  | 0.72 | 0.58~0.88 | 0.001 |
|  | 150~300 | 1187 | 7684 | 127(10.7%) |  | 0.46 | 0.36~0.59 | <0.001 |  | 0.69 | 0.53~0.91 | <0.001 |  | 0.71 | 0.52~0.96 | 0.025 |
|  | >300 | 2645 | 17356 | 221(8.4%) |  | 0.32 | 0.26~0.39 | <0.001 |  | 0.57 | 0.46~0.71 | <0.001 |  | 0.61 | 0.49~0.77 | <0.001 |
|  |  |  |  |  |  |  |  |  |  |  |  |  |  |  |  |  |
| **Recreational time(hour/day)** | <6 | 6571 | 44194 | 899(13.7%) |  | Ref |  |  |  | Ref |  |  |  | Ref |  |  |
|  | 6~8 | 2054 | 12580 | 378(18.4%) |  | 1.49 | 1.24~1.79 | <0.001 |  | 1.41 | 1.18~1.66 | <0.001 |  | 1.35 | 1.15~1.59 | <0.001 |
|  | >8 | 2288 | 13529 | 438(19.1%) |  | 1.51 | 1.29~1.78 | <0.001 |  | 1.63 | 1.41~1.90 | <0.001 |  | 1.55 | 1.34~1.79 | <0.001 |

y sample weights were taken into consideration in the Cox models accompanying the NHANES data. Covariates in Model 1 included VPA, MPA, recreational time, gender, age, race, marital status, educational level, family income level, smoke history. Model 2 also included albumin, creatinine levels and the presence of comorbidities including CHD, stroke, chronic bronchitis, liver conditions and high cholesterol. Abbreviations: HBP: hypertension; VPA: vigorous-intensity physical activity; MPA: moderate-intensity physical
